# Derivatives of 1-(2-Pyridyl)-3-pyrazolecarboxylic Acids as Ligands for Binding f-Elements

**DOI:** 10.3390/molecules31030541

**Published:** 2026-02-03

**Authors:** Olga I. Abramova, Alexey V. Ivanov, Nataliya E. Borisova, Victoriya A. Bitanova, Konstantin A. Lyssenko, Anastasiia V. Kharcheva, Svetlana V. Patsaeva

**Affiliations:** 1 Chemistry Department, Lomonosov Moscow State University, 119234 Moscow, Russiaharcheva.anastasiya@physics.msu.ru (A.V.K.); 2 Physics Department, Lomonosov Moscow State University, 119234 Moscow, Russia

**Keywords:** pyridylpyrazole, amide, f-elements, complexes, X-ray, luminescence

## Abstract

A series of amides of 1-(2-pyridyl)-5-arylpyrazole-3-carboxylic acids has been synthesized. Complexes of synthesized ligands with salts of 4f- and 5f-elements were obtained. The composition of the complexes in the gas phase, solution, and solid state has been studied. The luminescence parameters of europium complexes in solution have been determined.

## 1. Introduction

The chemistry of f-elements has been actively developing in recent decades. This is due to the wide range of applications of lanthanides (magnetic [1,2], optical [3,4], luminescent [5,6] materials, etc.), as well as the use of actinides as nuclear fuel. Therefore, the interest in new compoundscapable of forming stable complexes with f-elements is of high impact.

Among the potential ligands for spent fuel reprocessing technologies, substances containing various types of coordinating atoms should be especially highlighted. Thus, the most promising compounds appear to be those combining both “hard” (oxygen) and “softer” (nitrogen) donor centers within their structure. Such compounds include, for example, diamides of 2,6-pyridinedicarboxylic [7], 2,2′-bipyridyl-6,6′-dicarboxylic [8,9], and 2,9-phenanthrolinedicarboxylic acids [10].

When one of the pyridyl rings in 2,2′-bipyridyl is replaced by a pyrazole cycle, a new class of ligands is formed—pyridylpyrazoles. Such azole-based systems are also capable of forming complexes with f-elements [11]. One of their applications is the solvent extraction separation of actinide and lanthanide mixtures [12,13,14], making them promising materials for the industrial processing of liquid nuclear waste with high separation coefficients (in some cases, the separation factors for the Am^3+^/Eu^3+^ pair exceed 100) [15,16]. However, such ligands contain exclusively “soft” nitrogen metal binding sites. This leads to the formation of complexes with low stability constants [17,18], and the need to add a source of lipophilic anions (such as 2-bromohexanoate) to the extractant to carry out extraction by the mechanism of cation exchange [19,20].

The introduction of additional oxygen-containing functions can significantly enhance the stability of the resulting complexes. This possibility suggests the idea of obtaining pyrazolpyridine systems containing an acid function at position 3(5) of the pyrazole ring, which can be easily transformed into an amide function.

**Scheme 1:**
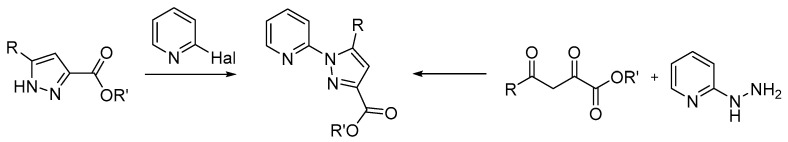
Approaches to the synthesis of 1-(2-pyridyl)pyrazole-3-carboxylates.

There are two main synthetic routes to obtain pyridylpyrazoles. The first involves the arylation of 3(5)-pyrazolecarboxylic acid derivatives using 2-halopyridines. The secondrouteentails the condensation of 2-pyridylhydrazines with 1,3-dicarbonyl compounds (Scheme 1). Thearylation of pyrazoles in the presence of bases is a well-known process [21,22,23]. Its main drawbackis the low selectivity in forming the target derivatives of 1-arylpyrazoles as a consequence of the tautomeric equilibrium in the starting compounds [24].

In the case of condensation of diketoesters with substituted hydrazines, a mixture of isomers can also be obtained [25,26]. However, their ratio is determined by the difference in the electrophilic activity of carbonyl groups, as well as the steric factors, which allows enhancing the reaction selectivity [27,28]. This paper presents the results of the synthesis of new 1-(2-pyridyl)pyrazole-3-carboxylic acid derivatives and the study of their complexes with f-elements.

## 2. Results

### 2.1. Synthesis of Ligands

**Scheme 2:**
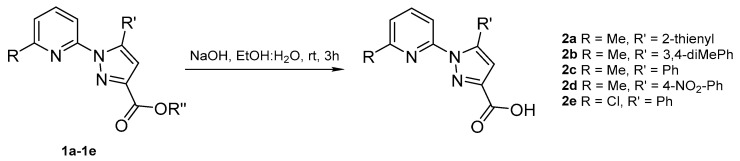
Hydrolysis of 1-(2-pyridyl)pyrazole-3-carboxylates.

Amides of 1-(2-pyridyl)pyrazole-3-carboxylic acids were chosen as the objects of this research. These compounds contain a complex of “soft” donor centers on nitrogen atoms and a “hard” binding center on an oxygen atom, which allows them to be used for REE (rare earth elements) binding. The choice of substituents in the pyrazole cycle was determined by the need to increase the selectivity of the condensation reaction of the initial diketoesters with pyridylhydrazine. Esters of pyrazole-3-carboxylic acids **1a**–**e** were synthesized, the hydrolysis of which led to the formation of the corresponding acids **2a**–**e** (Scheme 2).

**Scheme 3:**
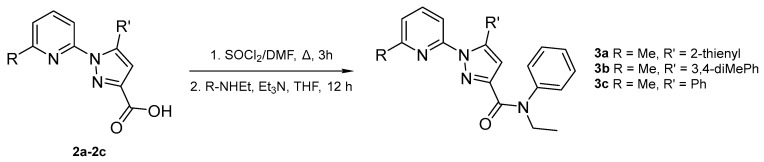
Synthesis of the amides of the 1-(2-pyridyl)pyrazole-3-carboxylic acids.

To ensure the solubility and increase the stability of ligands in an acidic extraction medium, amides **3a**–**c** were synthesized from acids **2a**–**c** (Scheme 3).

**Figure 1:**
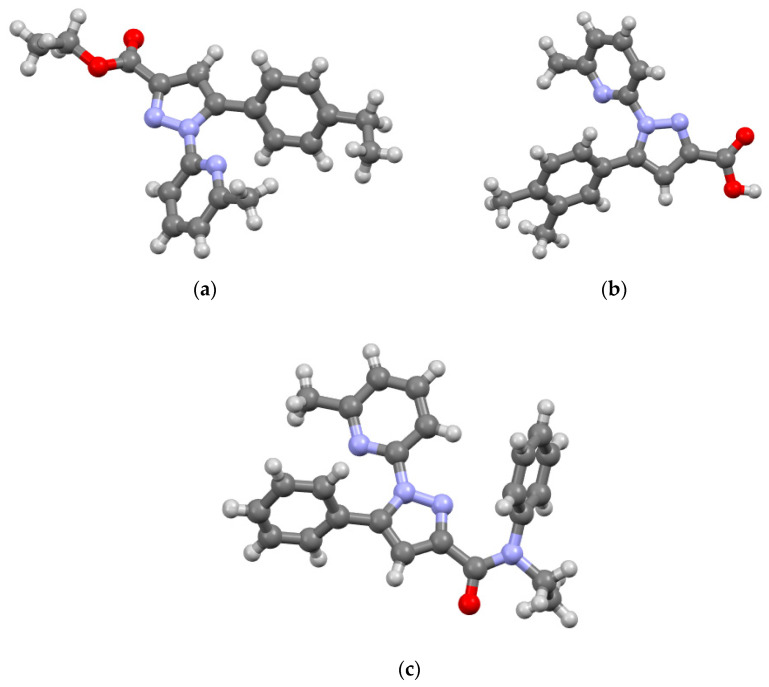
Structures of compounds **1f** (**a**), **2e** (**b**), and **3a** (**c**) (according to X-ray data).

This reaction proceeds with a high degree of selectivity, leading to the formation of the desired pyridylpyrazole isomer. ^1^H-NMR, ^13^C spectroscopy, MALDI-TOF, and X-ray data confirm the formation of the target products. The selectivity of the cyclization reaction with the formation of the desired isomers is confirmed by the X-raydiffraction data for ligand **3a**, as well as for intermediate compounds **1f** and **2e** (Figure 1).

Some interatomic distances and dihedral angles in the studied compounds are presented in Table 1 and Table 2, respectively. It can be seen that in all compounds the C=O groups lie in the plane of the pyrazole ring, while in both ether and amide the oxygen atom is in an anti-conformation with respect to the nitrogen atom in the pyrazole cycle. The pyridine ring is rotated relative to the pyrazole ring by ~130–140° (see Table 2),which is markedly different from the angle in bipyridylstructures, in which the dihedral angle between two nitrogen atoms in heterocycles is close to 180° [29]. The aryls in the fifth position of pyrazole ring are rotated relative to the plane of the heterocycle by 40–60°.

### 2.2. Complexes of Amide Ligands with REE

**Scheme 4:**
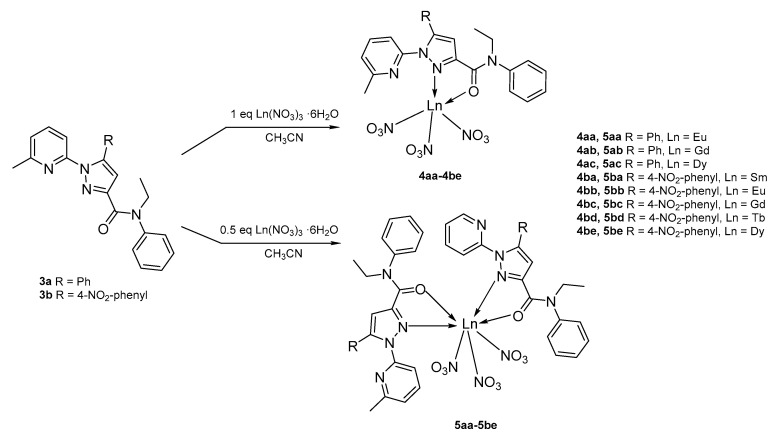
Synthesis of complexes of the obtained ligands with lanthanides.

**Figure 2:**
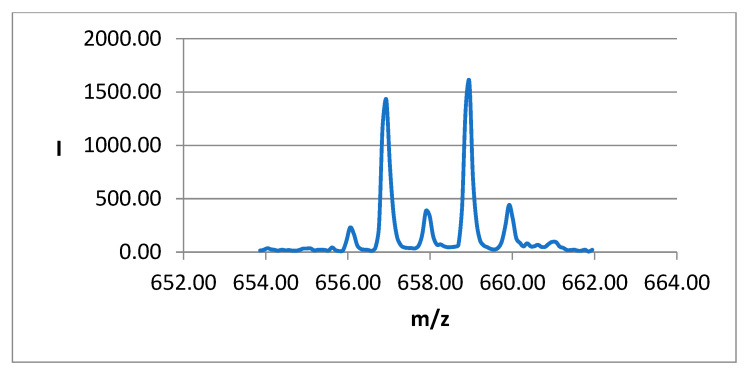
Mass spectrometric analysis of complex **4aa**. Peak [M-NO_3_]^+^.

The interaction of **3a**–**3b** amides with lanthanide nitrates in an acetonitrile medium leads to the formation of the corresponding complexes: **4aa**–**4be** in solution and in the gas phase, and **5aa**–**5be** in the solid phase (Scheme 4). Complexes with thorium nitrate (**4ad**, **5ad**, **4bf**, **5bf**) are formed in a similar way. The 1:1 composition of the metal–ligand complexes (in the gas phase) is confirmed by mass spectrometry (MALDI-TOF). The ionization of the complex is achieved by the release of one nitrate ion. In all cases, the isotopic distribution observed in the mass spectra corresponds to the theoretical one. A typical view of the mass spectrum is shown in Figure 2.

**Figure 3:**
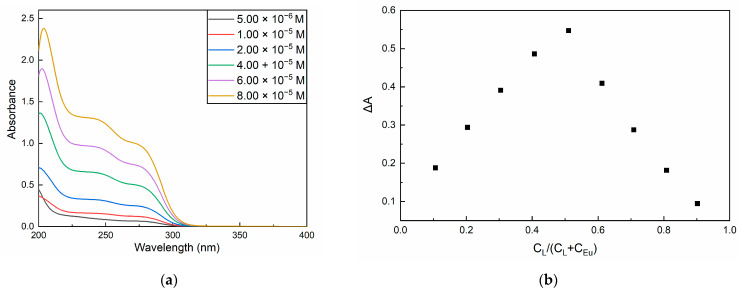
Absorption spectra of amide **3a** at different concentrations (**a**) and isomolar series for complex **4aa** (**b**).

The composition of the complexes in solution was studied by spectrophotometric methods. Using the Job’splot method, it was found that the **4aa** complex has a 1:1 metal–ligand composition in acetonitrile solution (Figure 3b).

The stability constant of the complex was calculated from the spectrophotometric titration data (Figure S1, see ESI). The spectrum data fitting yielded a stability constant value of lgβ = 4.39 ± 0.06. During the titration, the ligand spectrum changes very slightly, which complicates the analysis and may indicate low stability of the complexes formed. The absence of the EuL_2_ complex in the solution may be due to the insufficiently large size of the lanthanide ions for coordination with two ligand molecules. This result suggests that the ligand under study has the potential for stronger binding to actinide ions, which have larger ionic radii.

During the transition from the gas phase and solutions to the solid state, the composition of the resulting complexes changes dramatically. Their structure was originally determined based on the example of the **5aa** complex by the X-ray methods

**Figure 4:**
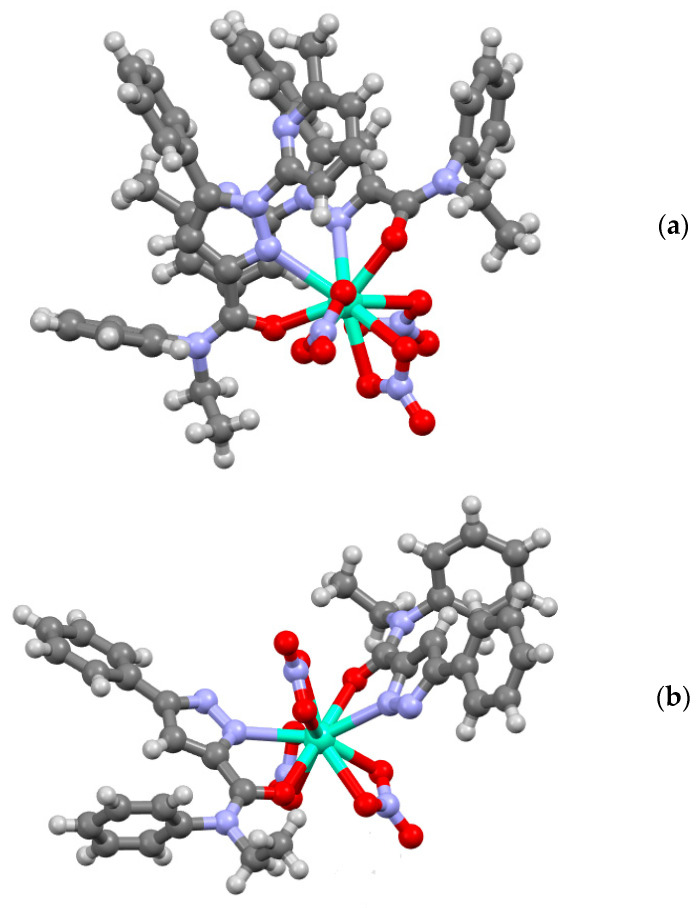
Structures of europium complexes **5aa** (with 1-(6-methylpyridine-2-yl)pyrazole, (**a**)) and **6** (with N–H pyrazole, (**b**)) (according to X-ray data).

According to X-ray diffraction data, the interaction of europium nitrate with ligand **3a** leads to the formation of the complex **5aa** with a metal–ligand composition of 1:2 in the solid phase (Figure 4a). The structure of the complexes is identical (see Table 1 and Table 2), which allows us to conclude that the composition and structure of the obtained substances of this class are constant. Let us look at it using the example of substance **5aa**. Gadolinium **5ab** and dysprosium **5ac** complexes have the same structure (see Table 1 and Table 2).

In this compound, the coordination polyhedron of the europium ion is a bicapped square antiprism. The coordination number of the metal in this complex is 10. Coordination is carried out with two oxygen atoms of the amide groups, two nitrogen atoms from the pyrazole rings, and oxygen atoms from the nitrate ions acting as bidentate ligands. The nitrogen atoms of the pyridine ring are not involved in metal binding; the pyridine cycle is rotated at an angle of ~130° relative to the plane of the pyrazole ring.

The complex **6** of europium trinitrate with N-ethyl-N,5-diphenyl-1H-pyrazole-3-carboxamide has the same composition (Figure 4b). The ligands form a sphenocorona coordination polyhedron around europium.

The coordination of ligands across the europium ion in this compound is carried out in a similar manner to that described for complexes 5. The dihedral angle of N–C–C–O is 9.7°, which is significantly less than in complexes of europium with 2,2′-bipyridyl-6, 6′-dicarboxylic acid diamides (20–30°) [9,29], but corresponds to the angle in complexes with phenanthroline ligands (~10°) [10,30]. The C=O bond (1.249 Å) is shortened compared to the starting carbonyls. The C_5_–C_6_ bond length (between pyrazole and the amide group) is 1.486 Å. The Eu–O and Eu–N bond lengths in the complex are 2.444 Å and 2.560 Å, respectively. In both complex compounds, an elongation of the O–C bond in the amide group is observed compared to the free ligand **3a** (1.231 A). In complex **5aa**, this bond turns out to be longer (1.257 Å) than in the complex with unsubstituted pyrazole **6** (1.249 Å). At the same time, the bond length between the amide group and pyrazole remains unchanged within the measurement accuracy. In complex **5aa,** the Eu–O bond is shorter (2.350 Å) than in complex **6** (2.444 Å). The opposite trend is observed for the Eu–N bonds (2.725 and 2.560 Å, respectively, Table 2).

It should be noted that the structure of the obtained complexes is quite unexpected. Pyridine is usually the stronger coordinating center. In this case, it is the pyrazole nitrogen that turns out to be the metal-binding center. Undoubtedly, this is due to the presence of an additional “hard” oxygen fragment of the amide group in the immediate vicinity of the pyrazole nitrogen.

### 2.3. Luminescent Studies

**Figure 5:**
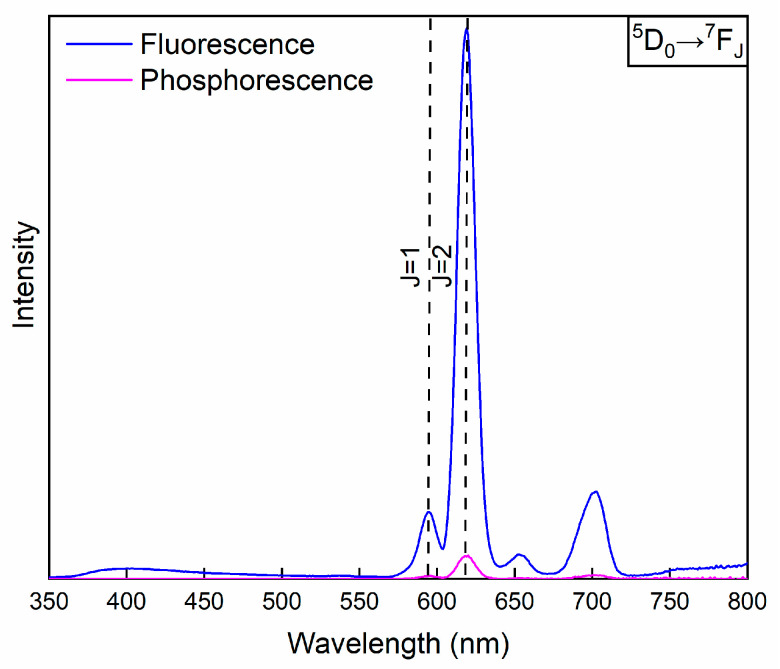
Luminescence emission spectrum of the **4aa** complex, λ_ex_ = 320 nm.

The luminescence emission spectra of complex **4aa** were analyzed. The resulting fluorescence spectrum (Figure 5) represents a characteristic set of narrow signals typical for europium complexes with organic ligands and has two main maxima: one centered at 592 nm, corresponding to the ^5^D_0_–^7^F_1_ transition, and another at 618 nm, corresponding to the ^5^D_0_–^7^F_2_ transition. The latter is the most intense peak in the spectrum. A broad, low-intensity signal corresponding to ligand fluorescence is observed in the 400 nm region. Phosphorescence is observed only for the complex, which confirms the low probability of this process for the organic ligand compared to non-radiative relaxation.

To determine the lifetime of the excited state, the phosphorescence decaykinetics of the complex was analyzed. The dependence of the radiation intensity on time was recorded at a wavelength of 618 nm, corresponding to the highest-energy transition ^5^D_0_–^7^F_2_. The decay kinetics is monoexponential, which indicates the presence of one unequal position occupied by the europium ion in the structure of the complex. The luminescence lifetime was found to be (1.05 ± 0.02) ms.

The quantum yield of luminescence was calculated using the reference dye method with rhodamine 6J as a standard:

(1) $$\Phi =\frac{I_{x}D_{et}}{D_{x}I_{et}}\Phi_{et}$$

where *I_x_* and *I_et_* are integrated luminescence intensities, *D_x_* and *D_et_* are optical densities of the sample and reference solutions at the excitation wavelength, and $\Phi_{et}$ is the reference luminescence quantum yield.

The quantum yield of luminescence was found to be 9.3%. The calculated value characterizes the efficiency of energy transfer from ligand levels to metal levels. The obtained result correlates with data indicating the low stability of the formed complex. Weak coordination between the europium ion and the ligand can lead to an increased probability of non-radiative relaxation, thereby reducing the quantum yield. A similar effect can be exerted by the presence of inorganic ligands, such as nitrate anions or water molecules, in coordinatively unsaturated complexes.

## 3. Materials and Methods

All reagents and solvents were obtained from commercial sources. Acetonitrile (99.95%, Biosolve BV (Biosolve Chemicals, Dieuze, France)) was dried over molecularsieves (zeolite KA, 3Å, balls, diameter 1.6–2.5 mm) prior to use. The water content was estimated as40 ± 5 ppm by Karl Fisher titration (Mettler Toledo, C20, coulometric KF titrator, Mettler-Toledo, Inc., Columbus, OH, USA). Lanthanide metal nitrates Ln(NO_3_)_3_·6H_2_O (purity > 99%) were storedin a closed container over silica gel balls. The stocksolutions of ligands and europium salts were preparedby weighing the amounts of the respective chemicalsand dissolving them in acetonitrile. A more detailed description of the syntheses is provided in the ESI (Synthetic procedures).

^1^H and ^13^C NMR spectra were recorded on a BrukerAvance-400 MHz NMR spectrometer (Bruker Corporation, Billerica, MA, USA) at 24 °C.

Preliminary purity testing of all organic compounds was performed using an NMReady 60 Pro spectrometer (Calgary, AB, Canada).

The CHN analysis was carried out using a CHNOS Elemental Analyzer Vario MICRO (Langenselbold, Germany: Elementar Analysensysteme GmbH).

The IR spectra were recorded on a Varian 640 FTIR spectrometer (Varian Medical Systems, Palo Alto, CA, USA). The spectra were recorded at a resolution of 4 cm^−1^, and the number of scans was 16.

Single crystals of **5aa**–**ac** and **6** were investigated on a Bruker D8 QUEST single-crystal X-ray diffractometer equipped with a PHOTON II detector, charge-integrating pixel array detector (CPAD), laterally graded multilayer (Goebel) mirror, and a microfocus Mo-target X-ray tube (λ = 0.73071 Å). The frame width of 0.5° was employed for data collection. Data reduction and integration were performedwith the Bruker software package SAINT (Version 8.40B) [31]. The data were corrected for Lorentz and polarization effects. The absorption correction was performed using the multi-scan routine as implemented in SADABS (Version 2016/2) [32]. Crystal structure solution and refinement were performed using the SHELX-2018 package [33]. Atomic positions were located using dual methods and refined using a combination of Fourier synthesisand least-squares refinement in isotropic and anisotropic approximations. All non-hydrogen atoms were refined with anisotropic displacement parameters. All C–H hydrogen atoms were placed in ideal calculated positions and refined as riding atoms with relative isotropic displacement parameters taken as *U*_iso_(H) = 1.5*U*_eq_(C) for methyl H atoms and *U*_iso_(H) = 1.2*U*_eq_(C) otherwise.

Massspectra were recorded using a MALDI-TOF Reflex 3 instrument (BRUKER, Bruker Corporation, Billerica, MA, USA) in the positive ion mode (UV laser, 337 nm) without the use of a matrix.

### 3.1. Spectrophotometry

Ultraviolet–visible (UV-vis) spectra were recorded at ambient temperature (24.5 ± 1.0 °C) in the wavelength region of 260–500 nm (1 nm interval) on a Hitachi U-1900 spectrophotometer (Hitachi, Tokyo, Japan) using 10 mm path-length quartz cells. The implementation of the Beer–Lambert law was determined for the ligand within the range 0.005–0.08 mM. The method of continuous variation determined the binding stoichiometry of one-step complex formation between two different molecules (Job’s plot). The solutions of ligand **3a** and europium salt were prepared at a concentrationof ≈0.1 mM. For the spectrophotometric titration, a solution of ligand **3a** was prepared ca. 30 µM. A titrant solution of Eu(NO_3_)_3_·6H_2_O (ca. 0.5mM) was prepared by dissolvingof a sample of europium nitrate hydrate in a solution of ligand **3a**. A 2 mL solution of ligand **3a** was titrated with 6 µL of Eu(NO_3_)_3_·6H_2_O. The kinetic experiments showed that the absorbance becomes stable within 1 min. The stability constant of the complex was calculated using the HypSpec2014 software package [9].

### 3.2. Luminescence

The luminescence emission spectra were recorded using a Hitachi F-7000 luminescence spectrometer (Hitachi, Tokyo, Japan) at room temperature. The 90° geometry was used for acetonitrile solutions placed in a standard quartz cuvette with an optical path length of 10 mm. The excitation wavelength was set at 320 nm. Registration was performed within the spectral region 350–800 nm with a spectral interval 0.2 nm. The luminescence excitation spectra were registered at 618 nm with excitation in the spectral range 250–600 nm with a spectral interval of 1 nm. The scan speed was 1200 nm/min, and the spectral slits (excitation monochromator and emission monochromator) were 5 × 5 nm. The PMT voltage was 400 V.

### 3.3. Preparation of Ligands

Diketoesters were obtained via condensation of the corresponding ketones with diethyl oxalate in the presence of a base. Esters **1a**–**e** were synthesized by condensation of pyridylhydrazines with the corresponding diketoesters. Pyrazole carboxylic acids **2a**–**e** were prepared by subsequent alkaline hydrolysis. Amides **3a**–**c** were synthesized via reaction of the pyrazolecarboxylic acid chlorides with N-ethylaniline.

### 3.4. Synthesis of Complexes with f-Elements Nitrates

Complexes **5aa**–**ac and 5ba**–**be** were obtained by reacting the corresponding amides with lanthanide trinitrates in acetonitrile. Complexes **5ad** and **5bf** were obtained similarly by interaction with thorium nitrate.

General procedure. The ligand (100 mg) was dissolved by heating in 2 mL of dry acetonitrile. An excess of the corresponding salt of the f-element was added to the solution. The solution was cooled to room temperature. The precipitate was filtered out, washed with cold acetonitrile (1–1.5 mL), and then dried in air to a constant mass.

**N-ethyl-1-(6-methylpyridin-2-yl)-N,5-diphenyl-1H-pyrazole-3-carboxamide europium trinitrate (5aa)**. Yield 31%. Massspectrum (MALDI-TOF), *m*/*z*: 658 ([L+Eu(NO_3_)_2_]^+^). Found (%): C 51.27, H 3.80, N 14.50. Calculated for C46H40N11O11Eu (%): C 51.40, H 3.75, N 14.33. IR (KBr, ν, sm^−1^): 1601 (CON), 1576 (C=N Py), 1493 (pyrazole), 1306 (NO_3_^−^).

**N-ethyl-1-(6-methylpyridin-2-yl)-N,5-diphenyl-1H-pyrazole-3-carboxamide gadolinium trinitrate (5ab).** Yield25%. Mass-spectrum (MALDI-TOF), *m*/*z*: 664([L+Gd(NO_3_)_2_]^+^). Found (%): C 50.98, H 3.77, N 14.11. Calculated for C_46_H_40_N_11_O_11_Gd (%): C 51.15, H 3.73, N 14.26. IR (KBr, ν, sm^−1^): 1612 (CON), 1581 (C=N Py), 1493 (pyrazole), 1306 (NO_3_^−^).

**N-ethyl-1-(6-methylpyridin-2-yl)-N,5-diphenyl-1H-pyrazole-3-carboxamide dysprosium trinitrate (5ac)**. Yield 28%. Massspectrum (MALDI-TOF), *m*/*z*: 670 ([L+Dy(NO_3_)_2_]^+^). Found (%): C 51.07, H 3.75, N 14.22. Calculated for C46H40N11O11Dy (%): C 59.90, H 3.71, N 14.20. IR (KBr, ν, sm^−1^): 1640 (CON), 1594 (C=N Py), 1573 (pyrazole), 1289 (NO_3_^−^).

**N-ethyl-1-(6-methylpyridin-2-yl)-N,5-diphenyl-1H-pyrazole-3-carboxamide thorium tetratrinitrate (5ad)**. Yield 25%. Massspectrum (MALDI-TOF), *m*/*z*: 800 ([L+Th(NO_3_)_2_]^+^). Found (%): C 45.82, H 3.49, N 13.57. Calculated for C_46_H_40_N_12_O_14_Th (%): C 45.40, H 3.31, N 13.81. IR (KBr): IR (KBr): 1602 (CON), 1580 (C=N Py), 1487 (pyrazole), 1297 (NO_3_^−^).

**N-ethyl-1-(6-methylpyridin-2-yl)-5-(4-nitrophenyl)-N-phenyl-1H-pyrazole-3-carboxamide samarium trinitrate (5ba)**. Yield 27%. Massspectrum (MALDI-TOF), *m*/*z*: 703([L+Sm(NO_3_)_2_]^+^). Found (%): C 47.21, H 3.24, N 15.25. Calculated for C_46_H_38_N_13_O_15_Sm (%): C 47.50, H 3.29, N 15.65. IR (KBr, ν, sm^−1^): 1607 (CON); 1580 (C=N Py); 1487 (pyrazole); 1517, 1324 (NO_2_); 1297 (NO_3_^−^).

**N-ethyl-1-(6-methylpyridin-2-yl)-5-(4-nitrophenyl)-N-phenyl-1H-pyrazole-3-carboxamide europium trinitrate (5bb)**. Yield 25%. Massspectrum (MALDI-TOF), *m*/*z*: 704 ([L+Eu(NO_3_)_2_]^+^). Found (%): C 46.99, H 3.22, N 15.17. Calculated for C_46_H_38_N_13_O_15_Eu (%): C 47.47, H 3.29, N 15.63. IR (KBr, ν, sm^−1^): 1613 (CON); 1576 (C=N Py); 1501 (pyrazole); 1520, 1330 (NO_2_); 1280 (NO_3_^−^).

**N-ethyl-1-(6-methylpyridin-2-yl)-5-(4-nitrophenyl)-N-phenyl-1H-pyrazole-3-carboxamide gadolinium trinitrate (5bc).** Yield 30%. Massspectrum (MALDI-TOF), *m*/*z*: 710 ([L+Gd(NO_3_)_2_]^+^). Found (%): C 47.08, H 3.18, N 15.24. Calculated for C_46_H_38_N_13_O_15_Gd (%): C 47.22, H 3.27, N 15.56. IR (KBr, ν, sm^−1^): 1609 (CON); 1582 (C=N Py); 1499 (pyrazole); 1524, 1340 (NO_2_); 1280 (NO_3_^−^).

**N-ethyl-1-(6-methylpyridin-2-yl)-5-(4-nitrophenyl)-N-phenyl-1H-pyrazole-3-carboxamide terbium trinitrate (5bd)**. Yield 33%. Massspectrum (MALDI-TOF), *m*/*z*: 710([L+Tb(NO_3_)_2_]^+^). Found (%): C 47.10, H 3.17, N 15.40. Calculated for C_46_H_38_N_13_O_15_Tb (%): C 47.15, H 3.27, N 15.54. IR (KBr, ν, sm^−1^): 1605 (CON); 1573 (C=N Py); 1504 (pyrazole); 1530, 1312 (NO_2_); 1280 (NO_3_^−^).

**N-ethyl-1-(6-methylpyridin-2-yl)-5-(4-nitrophenyl)-N-phenyl-1H-pyrazole-3-carboxamide dysprosium trinitrate (5be).** Yield 28%. Massspectrum (MALDI-TOF), *m*/*z*: 715 ([L+Dy(NO_3_)_2_]^+^). Found (%): C 47.12, H 3.12, N 15.52. Calculated for C_46_H_38_N_13_O_15_Dy (%): C 47.01, H 3.26, N 15.49. IR (KBr, ν, sm^−1^): 1609 (CON); 1582 (C=N Py); 1504 (pyrazole); 1541, 1320 (NO_2_); 1283 (NO_3_^−^).

**N-ethyl-1-(6-methylpyridin-2-yl)-5-(4-nitrophenyl)-N-phenyl-1H-pyrazole-3-carboxamide thorium trinitrate (5bf)**. Yield 29%. Mass-spectrum (MALDI-TOF), *m*/*z*: 845 ([L+Th(NO_3_)_2_]^+^). Found (%): C 41.88, H 3.06, N 15.12. Calculated for C_46_H_38_N_14_O_18_Th (%): C 42.27, H 2.93, N 15.00. IR (KBr, ν, sm^−1^): 1602 (CON); 1602 (C=N Py); 1497 (pyrazole); 1530, 1311 (NO_2_); 1291 (NO_3_^−^).

## 4. Conclusions

Thus, we have obtained new ligands for binding f-elements, which form stable metal–ligand 1:2 complexes in the solid phase. The luminescent properties of the complexes were also investigated.

## Figures and Tables

**Table 1:** Dihedral angles in compounds **1f**, **2e**, **3a**, **5aa**–**ac**, and **6**.

Tangle	Pyrazole-Pyridine	Pyrazole-Amide	Pyrazole-Phenyl	Met-O=C-C	Met-N_1_-C_1_-C_4_	Met-N_1_-C_1_-C_2_
**1f**	−140.5(1)	−153.8(1)	159.7(1)	-	-	-
**2e**	129.0(2)	144.0(2)	5.5(2)	-	-	-
**3a**	−140.5(1)	159.7(1)	50.7(2)	-	-	-
**5aa (Eu)**	136.3(2)	−18.4(3)	96.1(3)	30.5	1.1	−170.8
**5ab (Gd)**	136.2(1)	18.3(2)	96.6(2)	30.5	0.9	−170.66
**5ac (Dy)**	136.6(2)	−17.9(2)	97.7(2)	29.8	1.0	−170.2
**6 (Eu)**	136.3(2)	9.7(2)	−88.2(3)	30.5	1.1	−170.8

**Table 2:** Bond lengths in compounds **1f**, **2e**, **3a**, **5aa**–**ac**, and **6**.

Bond	C=O	C-(C=O)	N_2_-C_5_(Py)	Met-O=C	Met-N
**1f**	1.203(2)	1.480(2)	1.433(2)	-	-
**2e**	1.222(2)	1.474(2)	1.433(2)	-	-
**3a**	1.231(2)	1.489(2)	1.425(2)	-	-
**5aa (Eu)**	1.249(2)	1.486(3)	1.369 (3)	2.444	2.560
**5ab (Gd)**	1.257(1)	1.484(2)	1.440(2)	2.335	2.7076
**5ac (Dy)**	1.254(2)	1.486(3)	1.441(3)	2.300	2.689
**6 (Eu)**	1.257(2)	1.487(3)	1.449(4)	2.350	2.725

## Data Availability

The data presented in this study are available on request from the corresponding author.

## Supplementary Materials

The following supporting information can be downloaded at: https://www.mdpi.com/article/10.3390/molecules31030541/s1, File S1. Synthetic procedures, physical characteristics of the obtained compounds, spectrophotometric titration data, crystallographic parameters and final residuals for the single-crystal XRD experiments, crystallographic data. Figure S1. Spectrophotometric titration of ligand 3a with a solution of Eu(NO3)3 salt in acetonitrile. Table S1. Crystallographic parameters and final residuals for the single-crystal XRD.
