# Supplementary material for: Derivatives of 1-(2-Pyridyl)-3-pyrazolecarboxylic Acids as Ligands for Binding f-Elements
Source: Molecules. 2026 Feb 3;31(3):541. doi: 10.3390/molecules31030541 (PMC12899771; doi:10.3390/molecules31030541)
Supplement: Supplementary file 1 [file molecules-31-00541-s001.zip › SUPPLEMENTARY_Abramova+.pdf]

SUPPLEMENTARY

Synthetic procedures

Synthesis of diketoesters

Approach A

General procedure .A mixture of 0.1 mol ketone and 16 g (0.11 mol) diethyl oxalate in 60 ml methanol was added to the sodium methylate solution (obtained by dissolving 3 g of sodium in 60 ml methanol) with stirring. The reaction mixture was heated to precipitate and left overnight. The mixture was diluted with 200 ml of water. The formed sediment was filtered out. The resulting solid compound was placed in 150 ml of 25% hydrochloric acid and heated to a boil. After cooling the reaction mixture, the target diketoester was filtered and dried in air.

Ethyl 2,4-dioxo-4-phenylbutanoate. Yield 87%. ^{1}HNMR ($CDCl_3$): 1.4 (s, 3 H) 4.4 (q, 2 H) 7.1 (s, 1 H) 7.5 (t, 2 H) 7.6 (t, 1 H) 8.0 (d, 2 H)15.3 (brs, 1 H)

Ethyl 4-(4-nitrophenyl)-2,4-dioxobutanoate. Yield 71%.^{1}HNMR ($CDCl_3$) δ, ppm 1.4 (t, 3 H) 4.4 (q, 2 H) 7.1 (s, 1 H) 8.1 (d, 2 H) 8.4 (d, 2 H).

Methyl 2,4-dioxo-4-(thiophene-2-yl)butanoate. Yield 72%.^{1}H NMR ($CDCl_3$):δ ppm 3.93 (s, 3 H) 6.92 (s, 1H) 7.18 (t, 1 H) 7.75 (dd, 1 H) 7.85 (dd, 1 H) 14.3-14.7 (br s, 1 H).

Ethyl 4-(3,4-dimethylphenyl)-2,4-dioxobutanoate. Yield 73%.^{1}H NMR ($CDCl_3$): δ ppm 1.4 (t, 3 H) 2.2 (m, 3 H) 2.3 (m, 3 H) 4.4 (q, 2 H) 7.0 (s, 1 H) 7.2 (m, 1 H) 7.6 (m, 1 H) 7.9 (m, 1 H).

Approach B

General procedure. A mixture of 0.05 mol ketone and 0.055 mol diethyl oxalate was added to 1.2 g (0.052 mol) of finely crushed sodium in 100 ml of toluene at room temperature. The mixture was stirred for 1-1.5 hours and left overnight. 120 ml of water was added to the reaction mass. The aqueous layer was separated and washed with ether (2X20 ml). The organic fractions were discarded, the aqueous was acidified with acetic acid to pH 4. The target compound was extracted with methylene chloride (2X30 ml). The organic phases were dried with sodium sulfate. The solvent was removed in vacuum to obtain a pure diketoester.

Ethyl 4-phenyl-2,4-dioxobutanoate. Yield64 %.^{1}H NMR ($CDCl_3$):δ ppm(1.42 (t, *J*=7.14 Hz, 3 H) 4.40 (q, *J*=7.24 Hz, 2 H) 7.09 (s, 1 H) 7.45 - 7.56 (m, 3 H) 7.58 - 7.67 (m, 1 H) 7.89 - 8.07 (m, 2 H), 15.2 (br. S, 1H).

Ethyl 4-(4-ethylphenyl)-2,4-dioxobutanoate. Yield 50%.^{1}H NMR ($CDCl_3$):δppm 1.3 (m, 3 H) 1.4 (t, 3 H) 2.7 (m, 2 H) 4.4 (q, 2 H) 7.0 (s, 1 H) 7.3 (d, 2

H) 7.9 (d, 2 H). ^{13}C NMR ($CDCl_3$):δ ppm 14.1 (s, 1 C) 15.0 (s, 1 C) 15.1 (s, 1 C) 29.0 (s, 1 C) 62.6 (s, 1 C) 76.7 (s, 1 C) 77.0 (s, 1 C) 77.2 (s, 1 C) 77.4 (s, 1 C) 97.8 (s, 1 C) 128.0 (s, 1 C) 128.1 (s, 1 C) 128.1 (s, 1 C) 128.4 (s, 1 C) 128.5(s, 1 C)128.6 (s, 1 C)129.0(s, 1 C) 132.4 (s, 1 C)151.1(s, 1 C)162.3 (s, 1 C)169.2 (s, 1 C)190.7 (s, 1 C).

Preparation of pyridylhydrazines.

Generalprocedure.

4 g of 2-halopyridine was dissolved in isopropyl alcohol, and an excess of hydrazine hydrate (1.5 g) was added. The resulting mixture was boiled for 8 hours. At the end of the reaction, the solvent and excess hydrazine were removed in a vacuum. The resulting substance was extracted with methylene chloride, washed with a small amount of saturated potassium carbonate solution, water, and dried over potassium carbonate. The solvent was removed in vacuum to obtain the desired compound.

6-methyl-2-pyridylhydrazine. Yield75%. M. p. 54-56ºC.^1H NMR ($CDCl_3$):δ ppm 2.7 (dd, 3 H) 6.5 (m, 1 H) 6.9 (m, 1 H) 7.2 (m, 1 H).

6-chloro-2-pyridylhydrazine. Yield 77%. ^1H NMR ($CDCl_3$) δ ppm: 6.6 (dd, 1 H) 6.7 (dd, 1 H) 7.4 (dd, 1 H).

Preparation of esters of 5-substituted 1-arylpyrazole-3-carboxylic acids.

General procedure.

0.01 mol of the corresponding diketoester and pyridylhydrazine were dissolved in 15-20 ml of glacial acetic acid. The resulting solution was boiled for 2-3 hours. After cooling, the precipitate was filtered, rinsed with water and dried in air.If no precipitation occurred, the acetic acid was removed in the vacuum of a water jet pump, the remainder was rinsed with water and dried in air.

Ethyl 1-(6-methylpyridine-2-yl)-5-phenyl-1H-pyrazole-3 carboxylate (1a). Yield 65%.^1H NMR ($CDCl_3$):δ ppm 1.4 (s, 3 H) 2.4 (s, 3 H) 4.4 (q, 2 H) 7.0 (s, 1 H) 7.1 (s, 1 H) 7.2 (s, 3 H) 7.3 (s, 1 H) 7.3 (s, 2 H) 7.6 (s, 1 H).

Methyl 1-(6-methylpyridine-2-yl)-5-(4-nitrophenyl)-1H-pyrazole-3 carboxylate (1b). Yield 64%.^1H NMR ($CDCl_3$): δ ppm 2.7 (s, 3 H) 3.9 (s, 3 H) 6.7 (d, 1 H) 7.1 (d, 1 H) 7.1 (s, 1 H) 7.6 (d, 1 H) 7.8 (t, 2 H) 8.2 (d, 2 H).

Ethyl 1-(6-chloropyridine-2-yl)-5-phenyl-1H-pyrazole-3 carboxylate(1c). Yield 60%. ^1H NMR ($CDCl_3$):δ ppm 1.4 (s, 3 H) 4.5 (s, 2 H) 7.0 (s, 1 H)7.3 (s, 3 H) 7.4 (s, 3 H) 7.6 (s, 1 H) 7.7 (s, 1 H).

Methyl 1-(6-methylpyridine-2-yl)-5-(2-thiophene)-1H-pyrazole-3-carboxylate (1d). Yield 52%. ^{1}H NMR ($CDCl_3$): δ ppm 2.5 (s, 3 H) 3.7 (s, 3 H) 6.9 (t, 1 H) 7.01 (d, 1 H) 7.07 (s, 1 H) 7.2 (d, 1 H) 7.3 (m, 2 H) 7.7 (t, 1 H).

Methyl1-(6-methylpyridine-2-yl)-5-(3,4-dimethylphenyl)-1H-pyrazole-3-carboxylate (1e). Yield 73%. ^{1}H NMR ($CDCl_3$):δ ppm 2.2 (s, 3 H) 2.3 (s, 3 H) 2.7 (m, 3 H) 3.9 (s, 3 H) 6.7 (m, 1 H) 7.1 (m, 1 H) 7.1 (m, 1 H) 7.1 (s, 1 H) 7.4 (m, 1 H) 7.6 (m, 1 H) 7.8 (m, 1 H).

Ethyl 1-(6-methylpyridine-2-yl)-5-(4-ethylphenyl)-1H-pyrazole-3 carboxylate (1f). Yield 67%.^{1}H NMR ($CDCl_3$): δ ppm 1.2 (t, 3 H) 1.4 (t, 3 H) 2.4 (s, 3 H) 2.6 (m, 2 H) 4.4 (q, 2 H) 7.0 (s, 1 H) 7.1 (q, 5 H) 7.2 (d, 1 H) 7.6 (t, 1 H). ^{13}C NMR ($CDCl_3$): δ ppm 13.9 (s, 1 C)14.9 (s, 1 C)23.5 (s, 1 C) 28.1 (s, 1 C) 52.9 (s, 1 C) 60.6 (s, 1 C) 76.4 (s, 1 C) 76.6 (s, 1 C) 76.8 (s, 1 C) 109.5 (s, 1 C) 116.3 (s, 1 C) 122.5 (s, 1 C) 126.7 (s, 1 C) 127.2 (s, 1 C) 128.3 (s, 1 C) 137.9 (s, 1 C) 144.1 (s, 1 C) 144.3 (s, 1 C) 144.8 (s, 1 C) 150.8 (s, 1 C) 157.8 (s, 1 C) 161.9(s, 1 C).

Hydrolysis of esters 1a-1e.

General procedure.

The ester (0.05–0.1 mol) was dissolved in a mixture of water and ethyl alcohol in a ratio of 1:1. An excess of 15% sodium hydroxide solution was added to the resulting solution and left overnight. The formed precipitate was filtered, dissolved in water and acidified with acetic acid to pH = 3-4. The precipitate was filtered and dried in air.

1-(6-methylpyridine-2-yl)-5-phenyl-1H-pyrazole-3-carboxylic acid (2a). Yield 70%.^{1}HNMR (DMSO-D6):δ ppm 2.4 (s, 3 H) 7.1 (s, 1 H) 7.2 (s, 1 H) 7.3 (s, 2 H) 7.3 (s, 4 H) 7.7 (s, 1 H).

1-(6-methylpyridine-2-yl)-5-(4-nitrophenyl)-1H-pyrazole-3-carboxylic acid (2b). Yield 62.5%. ^{1}H NMR (DMSO-D6):δ ppm 2.3 (s, 3 H) 7.2 (s, 2 H) 7.3 (s, 1 H) 7.5 (s, 2 H) 7.8 (s, 1 H) 8.2 (s, 2 H).

1-(6-chloropyridine-2-yl)-5-(phenyl)-1H-pyrazole-3-carboxylic acid (2c). Yield 83%. ^{1}H NMR (DMSO-D6):δ ppm 7.1 (s, 1 H) 7.2 (s, 2 H) 7.3 (s, 3 H) 7.6 (s, 1 H) 7.7 (s, 1 H) 8.1 (s, 1 H).

1-(6-methylpyridine-2-yl)-5-(2-thiophene)-1H-pyrazole-3-carboxylic acid (2d). Yield 56%. ^{1}HNMR (DMSO-D6):δ ppm 2.2 (s, 3 H) 6.7 (s, 4 H) 6.8 (s, 1 H) 7.0 (s, 2 H). IR(ν, см$^{-1}$): 1710 (-COOH).

1-(6-methylpyridine-2-yl)-5-(3,4-dimethylphenyl)-1H-pyrazole-3-carboxylic acid (2e). Yield76%. IR (ν, см-1): 1710 (-COOH).

Amides of pyrazolecarboxylic acids 2a-2c.

General procedure.

The corresponding acids (5 mmol) were placed in a round-bottomed flask, an excess of thionyl chloride and a drop of DMF were added. The resulting mixture was boiled for 2-3 hours with a return refrigerator. After the end of the reaction, thionyl chloride was distilled at reduced pressure. The resulting substance was dissolved in THF and slowly added to a previously prepared solution of N-ethylaniline with an excess of triethylamine in THF. The mixture was boiled for 12 hours. After cooling, an equal volume of water was added to the solution and the organic layer was separated. The aqueous fraction was extracted with diethyl ether. The organic extracts were washed with water and dried over sodium sulfate. After removing the solvent in vacuum, the target compound was obtained.

N-ethyl-1-(6-methylpyridine-2-yl)-N,5-diphenyl-1H-pyrazole-3-carboxoamide (3a). Yield 60%.^{1}H NMR (DMSO-D6) d ppm 1.3 (s, 3 H) 2.2 (s, 3 H) 4.0 (s, 2 H) 7.0 (s, 1 H) 7.1 (s, 3 H) 7.2 (s, 4 H) 7.3 (s, 1 H) 7.3 (s, 4 H) 7.6 (s, 1 H).^{13}C NMR (DMSO-d_6):δ ppm 13.21 (s, 1 C)23.69 (s, 1 C) 45.12 (s, 1 C) 110.08 (s, 1 C) 115.66 (s, 1 C) 123.10 (s, 1 C) 127.58 (s, 1 C) 128.42 (s, 1 C) 128.66 (s, 1 C) 128.72 (s, 1 C) 128.85 (s, 1 C) 129.50 (s, 1 C) 129.61 (s, 1 C) 129.94 (s, 1 C) 130.50 (s, 1 C) 139.64 (s, 1 C) 142.98 (s, 1 C) 143.55 (s, 1 C) 147.76 (s, 1 C) 151.36 (s, 1 C) 157.20 (s, 1 C) 162.45.

N-ethyl-1-(6-methylpyridine-2-yl)-5-(4-nitrophenyl)-N-phenyl-1H-pyrazole-3-carboxoamide (3b). Yield 65%.^{1}HNMR (DMSO-D6) δppm 1.2 (m, 3 H) 2.1 (m, 3 H) 4.0 (m, 2 H) 7.0 (m, 1 H) 7.3 (m, 5 H) 7.4 (m, 4 H) 7.6 (s, 1 H) 8.1 (m, 2 H).13C NMR (151 MHz, Solvent) δ ppm 13.62 ,24.26 ,44.54 ,107.64 ,116.34 ,117.87 ,124.31 ,124.31 ,129.23 ,129.43 ,129.43 ,129.78 ,129.78 ,130.61 ,130.90 ,130.90 ,138.95 ,142.11 ,145.88 ,146.80 ,149.27 ,151.38 ,157.07 ,164.45

1-(6-chloropyridine-2-yl)-N-ethyl-N,5-diphenyl-1H-pyrazole-3-carboxoamide (3c). Yield 62.5%.^{1}HNMR (DMSO-D6):δppm 1.2 (m, 3 H) 3.9 (s, 2 H) 7.0 (m, 2 H) 7.2 (m, 7 H) 7.3 (m, 3 H) 7.7 (m, 1 H).^{13}C NMR ($CDCl_3$):δ ppm 13.62 (s, 1 C) 44.54 (s, 1 C) 106.74 (s, 1 C) 115.90(s, 1 C) 118.17 (s, 1 C) 126.77 (s, 1 C) 128.00 (s, 1 C) 129.23 (s, 1 C) 129.43 (s, 1 C) 129.43 (s, 1 C) 129.78 (s, 1 C) 129.78 (s, 1 C) 129.81 (s, 1 C) 129.81 (s, 1 C) 130.07 (s, 1 C) 130.07 (s, 1 C) 140.10 (s, 1 C) 142.11 (s, 1 C) 143.18 (s, 1 C) 149.47 (s, 1 C) 151.46 (s, 1 C) 152.94 (s, 1 C) 164.45.

Spectrophotometric titration data

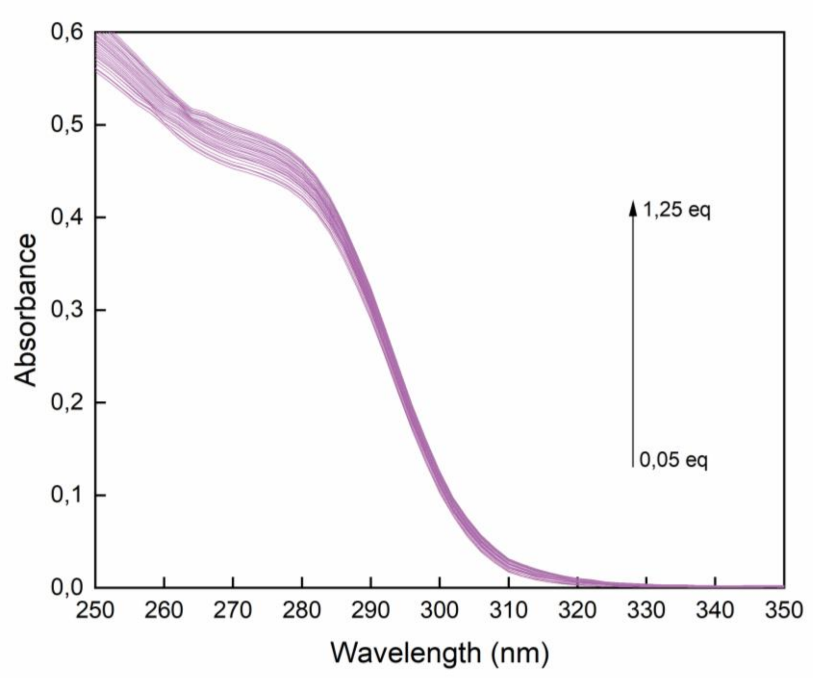

Figure S1. Spectrophotometric titration of ligand **3a** with a solution of $Eu(NO_3)_3$ salt in acetonitrile

XRD experiment data

Crystallographic parameters and final residuals for the single-crystal XRD experiments are given in Table S1.

Table S1. Crystallographicparameters and final residuals for the single-crystal XRD. experiments

	**2e**	**5ac (Dy)**	**5aa (Eu)**	**5ab (Gd)**	**6**
**CCDC**	2512829	2512826	2512827	2512828	2512825
**Formula**	$C_{18}H_{17}N_3O_2$	$C_{48}H_{44}DyN_{11}O_{11}$	$C_{48}H_{44}EuN_{11}O_{11}$	$C_{48}H_{44}GdN_{11}O_{11}$	$C_{38}H_{35}EuN_{10}O_{11}$
**Mw**	307.34	1113.44	1102.90	1108.19	959.72
**T, K**	120	110	110	110	100
**Crystal system**	Monoclinic				
**Space group**	C2/c	C2/c	C/2c	C2/c	C2/c
**Z(Z')**	4(0.5)	4(0.5)	4(0.5)	4(0.5)	4(0.5)
**a, Å**	16.974(2)	20.6931(9)	20.8549(7)	20.7801(5)	17.6240(4)
**b, Å**	12.3593(15)	14.9916(6)	15.0356(5)	15.0118(4)	18.9079(5)
**c, Å**	7.2891(9)	17.4679(8)	17.4959(10)	17.4674(4)	13.5574(4)
**α, °**	90	90	90	90	90
**β, °**	95.073(3)	117.6067(12)	117.6550(10)	117.6650(10)	117.4620(10)

**γ, °**	90	90	90	90	90
**V, Å^3**	1523.2(3)	4802.0(4)	4859.4(4)	4826.0(2)	4008.69(19)
**d_{calc}, g/cm^{-3}**	1.340	1.540	1.508	1.525	1.590
**□, cm^{-1}**	0.9	16.29	13.63	14.47	16.38
**F(000)**	648	2252	2240	2244	1936
**2□$_{max}$, °**	60	60	60	60	61
**Reflections collected**	19256	38057	42662	260944	22252
**Reflections unique**	4426	7005	7089	7040	6133
**Reflections with I > 2σ(I)**	2855	6583	6160	6822	5771
**Variables**	215	324	324	324	276
**R1**	0.0507	0.0236	0.0292	0.0148	0.0223
**wR2**	0.1584	0.0621	0.0671	0.0360	0.0516
**GOF**	0.927	1.065	1.028	1.089	1.073
**Largest differencein peak / hole (e/Å^3)**	0.342/-0.232	1.205/-0.962	1.209/-0.410	0.441/-0.745	1.698/-0.613
